# Autistic Traits, Empathizing–Systemizing, and Gender Diversity

**DOI:** 10.1007/s10508-021-02251-x

**Published:** 2022-04-25

**Authors:** Olivia Hendriks, Yimeng Wei, Varun Warrier, Gareth Richards

**Affiliations:** 1 School of Psychology, Faculty of Medical Sciences, Newcastle University, Dame Margaret Barbour Building, Wallace Street, Newcastle upon Tyne, NE2 4DR UK; 2 Autism Research Centre, Department of Psychiatry, University of Cambridge, Douglas House, 18b Trumpington Road, Cambridge, UK

**Keywords:** Autism, Empathizing–systemizing, Extreme male brain theory, Gender diversity, Transgender

## Abstract

Previous research indicates a link between autism and transgender and gender-diverse identities, though the association is not yet fully understood. The current study examined autistic traits (Autism Spectrum Quotient [AQ]), empathizing (Empathizing Quotient-Short [EQ-S]), and systemizing (Systemizing Quotient-Short [SQ-S]) in a sample of 89 adults and aimed to test whether gender-diverse individuals exhibit cognitive profiles consistent with predictions derived from the Extreme Male Brain (EMB) theory. As most research has considered only cisgender people, we recruited a more diverse sample by contacting > 200 UK LGBTQ+ organizations and posting on social media. A range of non-cisgender identities (e.g., transgender male, transgender female, non-binary, genderqueer, transmasculine) and non-heterosexual orientations (e.g., bisexual) were represented, and participants were categorized into one of four groups: (1) assigned female at birth but does not identify as female (transgender AFAB) (*n* = 32), (2) cisgender female (*n* = 21), (3) assigned male at birth but does not identify as male (transgender AMAB) (*n* = 18), and (4) cisgender male (*n* = 18). After controlling for age and autism diagnostic status, transgender AFAB participants had marginally higher AQ scores, and significantly higher SQ-S and systemizing-relative-to-empathizing (D) scores, compared with the cisgender female group. No such differences were detected between the transgender AMAB and cisgender male groups. Our findings are broadly in line with predictions derived from the EMB theory, though as no transgender AFAB participants reported being heterosexual, it was not possible to determine whether these effects relate specifically to gender identity, to sexual orientation, or to both.

## Introduction

Gender identity is distinct from the sex assigned at birth (typically male or female) and represents a person’s sense of their own gender. A person who is cisgender has a gender identity that is the same as the sex assigned at birth. There is a range of gender identities, including transgender, non-binary, gender fluid, bigender (and many others). In the current paper, we follow the definition of Warrier et al., (2020, p. 1) and refer to these and other diverse gender identities as “transgender and gender-diverse,” i.e., “individuals whose gender does not always correspond to the sex they were assigned at birth.” Gender dysphoria, on the other hand, is a strong and persistent dissonance between the sex assigned at birth and one’s gender identity (American Psychiatric Association, 2013). Recent evidence suggests although gender dysphoria is quite rare, people who self-identify as transgender or gender diverse represent a large and increasing percentage of the general population (Zhang et al., 2020). Furthermore, whereas gender dysphoria was previously found to affect relatively more birth-assigned males than birth-assigned females (Zucker & Lawrence, 2009), this trend appears to have reversed in recent adolescent cohorts, with relatively more birth-assigned females than birth-assigned males now presenting at gender clinics (Aitken et al., 2015; Zucker, 2017).

An emerging literature has examined the co-occurrence between autism/autistic traits and gender diversity and gender dysphoria (for recent reviews, see Glidden et al., 2016; Øien et al., 2018; Sala et al., 2020; van der Miesen et al., 2016). Autism spectrum conditions are characterized by unusually routine behavior, circumscribed interests, sensory hypersensitivity, difficulties adjusting to unexpected change, and social and communication problems (American Psychiatric Association, 2013), whereas autistic traits are those traits that are related to autism and found approximately normally distributed throughout the general population (Baron-Cohen et al., 2001; Ruzich et al., 2015). Autism spectrum conditions and autistic traits exhibit marked sex differences, with approximately three to four males diagnosed as autistic for every one female (Baio et al., 2018; Fombonne, 2009; Hull et al., 2020; Loomes et al., 2017). Research generally points to there being an overrepresentation of autism diagnoses (de Vries et al., 2010; Leef et al., 2019) and elevated autistic traits (Skagerberg et al., 2015) in children and adolescents presenting at gender clinics, and an elevated likelihood of diagnosed gender dysphoria (Hisle-Gorman et al., 2019) and parent-reported gender variant identity (Strang et al., 2014) in autistic children and adolescents. Autistic adolescents have also been shown to report a higher likelihood of a desire to be a different gender to that assigned at birth compared with general population adolescents (van der Miesen et al., 2018).

Until recently, the literature at the intersection between gender diversity and autism in adults was fairly limited (Glidden et al., 2016). However, Warrier et al. (2020) reported an analysis of five independently recruited cross-sectional datasets (*N* = 641,860) that showed higher rates of autism diagnosis in transgender and gender-diverse adults compared to cisgender controls. These findings are consistent with observations of an increased prevalence of autism diagnosis (Heylens et al., 2018) and elevated levels of autistic traits in adults attending gender clinics (Heylens et al., 2018; Jones et al., 2012; Pasterski et al., 2014), although it should be noted that not all studies have observed such effects (e.g., Vermaat et al., 2018; see also Nobili et al., 2018). Warrier et al. (2020) also showed that transgender and gender-diverse adults scored higher on a self-report measure of autistic traits, and other researchers have reported elevated levels of autistic traits in adults with self-reported gender diversity (Kristensen & Broome, 2015; Kung, 2020). Other studies have indicated that autistic adults report elevated rates of gender variance (Cooper et al., 2018; George & Stokes, 2018) as well as an elevated likelihood of desiring to be a different gender to that assigned at birth (van der Miesen et al., 2018).

The association between autism (and autistic traits) and gender diversity is gaining clinical and research interest (Glidden et al., 2016; Sala et al., 2020; Strang et al., 2018a, 2020; van der Miesen et al., 2016), and attention to the specific needs of those who are both autistic and have transgender and gender-diverse identities (and/or gender dysphoria) appear to be warranted. Although many transgender and gender diverse people do not need nor desire therapeutic support, it is worth noting that individuals who are either autistic (Griffiths et al., 2019; Hollocks et al., 2019) or gender diverse (Millet et al., 2017; Valentine & Shipherd, 2018) are at increased risk of a range of negative outcomes such as stress, isolation, poor self-esteem, anxiety, depression, alcohol/drug addiction, and suicidality. Additionally, the status of being a minority within a minority that is true of autistic people who are transgender or gender diverse is likely to result in even greater challenges (Coleman-Smith et al., 2020).

It has been suggested that the presentation of autism in many cisgender girls and women differs from that of cisgender boys and men (Bargiela et al., 2016; Dworzynski et al., 2012; Lai et al., 2017; see also Strang et al., 2020), and that people’s gender diversity is sometimes questioned because of their autism diagnosis (Coleman-Smith et al., 2020; Strang et al., 2018b). Efficacy of care for autistic individuals who choose to seek treatment for gender dysphoria may additionally be affected by some of the core features of autism. For instance, it could be that autistic individuals attending gender clinics experience increased difficulty in communicating their current needs to medical practitioners (Strang et al., 2018a). Such ideas suggest that a better understanding of the association between autism and gender diversity is necessary to provide more effective support when it is required.

An idea of potential relevance to explaining the connection between transgender and gender-diverse identities and the autism spectrum is that of Empathizing–Systemizing (E-S) theory (see Baron-Cohen, 2003). It posits that two constructs (empathizing and systemizing) underly human cognitive sex differences, and the Extreme Male Brain (EMB) theory (Baron-Cohen, 2002; Greenberg et al., 2018) extends this to the idea that autism is characterized by an exaggerated form of the male-typical cognitive profile. Empathizing is the ability to identify and respond appropriately to emotions and thoughts in others, whereas systemizing is the drive to analyze and build systems based on input-operation-output rules. Evidence for the veracity of these theories is provided by observations that, on average, non-autistic males score higher than non-autistic females on systemizing (*d* = 0.31), that non-autistic females score higher than non-autistic males on empathizing (*d* = 0.39), and that autistic males and females score higher in systemizing (males: *d* = 0.30; females: *d* = 0.39) and lower in empathizing (males: *d* = 0.41; females: *d* = 0.51) than non-autistic sex-matched controls (Greenberg et al., 2018). Sex differences observed for these measures also appear attenuated in autistic adults compared to non-autistic adults (Baron-Cohen et al., 2014; Greenberg et al., 2018). The difference in standardized empathizing and systemizing (D-score) is higher in non-autistic males than non-autistic females (*d* = 0.94), and higher in autistic males and females than non-autistic males and females, respectively (males: *d* = 1.34; females: *d* = 1.48) (Baron-Cohen et al., 2014 [present authors' calculations]). Interestingly, D-scores have been reported to explain 19 times more variance in autistic traits (43%) than other demographic variables, including sex (Greenberg et al., 2018), yet have typically not been examined in studies that investigate associations between autism and gender diversity.

Jones et al. (2012) were the first to suggest that predictions about the association between autism and transgender identity could be derived from the EMB theory. Elevated exposure to fetal androgens is posited to increase the likelihood of autism (Baron-Cohen et al., 2004, 2015) as well as a male gender identity (Hines, 2004); thus it can be predicted that autism and transgender identity in birth-assigned females will co-occur at a rate greater than chance (see also van der Miesen et al., 2016). In line with this prediction, Jones et al. (2012) reported that transgender males had higher levels of autistic traits than cisgender females and that transgender females and cisgender males did not differ in this regard. Di Ceglie et al. (2014) then reported that empathizing in adolescent transgender males and transgender females was lower than that of cisgender females, though they did not find any differences for systemizing. Although findings in this area have been equivocal (for a review, see van der Miesen et al., 2016), Stagg and Vincent (2019) and Kung (2020) recently confirmed the pattern of findings that would be predicted by the EMB theory: in both studies, lower empathizing and higher systemizing were found in transgender males relative to cisgender females, but no significant differences were found between transgender females and cisgender males. Although Warrier et al. (2020) reported lower empathizing and higher systemizing in transgender and gender-diverse individuals compared with cisgender individuals in a very large sample, no analysis by birth-assigned sex was possible in that study.

So far, relatively few studies have examined gender identities other than those that fit a binary classification (i.e., transgender males, transgender females, cisgender males, and cisgender females) in relation to autism/autistic traits. Kristensen and Broome (2015) examined a range of self-reported gender identities (e.g., androgyne, trans, transsexual, third gender), and reported that autistic traits were particularly elevated in individuals identifying as gender-queer (i.e., a person who does not subscribe to the conventional gender distinctions of male or female, but identifies as neither, both, or a combination of male and female genders). Furthermore, autistic individuals are more likely than neurotypical individuals to report their gender identity as atypical (i.e., not congruent with their birth sex) (Bejerot & Eriksson, 2014; George & Stokes, 2018). Self-identified non-binary individuals, on average, also score higher on self-report measures of autistic traits and systemizing, and lower on self-report measures of empathizing (Kung, 2020; Stagg & Vincent, 2019).

The present study aims to address gaps in the literature regarding how traits related to autism are associated with gender diversity by sampling a broad range of gender identities. Instead of comparing only transgender males and transgender females (or non-binary) participants with cisgender males and cisgender females, we used an online survey, and hypotheses derived from the EMB theory, to investigate the relationship between various transgender and gender-diverse identities and autistic traits, empathizing, and systemizing. Fundamentally, the E-S and EMB theories are about cognition along two axes (empathizing and systemizing) that impact behavior. The underlying mechanisms that shape this cognition are diverse and include genetics, prenatal hormones, and postnatal experience. Notably, many of these factors also underlie those associated with autism, a condition characterized by differences in cognition. Thus, the E-S theory and, its extension, the EMB theory suggest that the mechanisms underlying normative sex differences (i.e., those factors which give rise to sex differences in empathizing and systemizing) contribute, at least in part, to likelihood for autism, with empirical support emerging from self- and parent-report measures of these two axes. Both sex differences and rates of autism are different in transgender and gender-diverse populations. However, we again emphasize that the mechanisms underlying gender identity are equally complex. As such, one prediction emerging from the E-S and EMB theories is that transgender and gender-diverse individuals will score differently on these two axes of cognition compared to cisgender individuals. Considering this alongside the more specific hypotheses derived from EMB theory by Jones et al. (2012) and Nobili et al. (2018), we predicted that, compared to cisgender females, transgender AFAB participants would have more male-typical scores on traits associated with autism. More precisely, we predicted that transgender AFAB participants would have higher scores for autistic traits (AQ) and systemizing (SQ-S), lower scores for empathizing (EQ-S), and a higher systemizing-relative-to-empathizing (D) score, and that there would be no significant differences on these variables between transgender AMAB individuals and cisgender males. Given that the E-S and EMB theories make no predictions on sex vis-à-vis gender, we hesitate to speculate regarding possible differences between groups of gender-diverse individuals and hence do not test if transgender AFAB and transgender AMAB individuals score differently to each other.

## Method

### Participants

To ensure that a broad range of gender identities were represented, we contacted > 200 lesbian, gay, bisexual, transgender, queer + (LGBTQ+) support groups across the UK via email to ask for assistance in advertising our survey via their social media channels. The email explained that the study would take approximately 20 min, that it would examine gender identity, personality, and prenatal testosterone exposure, and that it was being conducted as part of an M.Sc. dissertation project at Newcastle University. We further clarified that the study had been granted approval by Newcastle University, and provided the following as a suggested message to share on social media: “Interested in gender and personality? Please consider taking part in a 20-minute online survey for a chance to win a £25.00 Amazon voucher. Please follow this link if you would like to take part:” (followed by the survey link). To ensure we enlisted similar numbers of cisgender and gender-diverse participants, and that each of the four groups (cisgender females, cisgender males, transgender AFAB, transgender AMAB) examined was represented by a broad range of sexual orientations, we also recruited participants via online survey exchanges (e.g., SurveyCircle, PollPool) and through personal contacts. No exclusion criteria were specified other than that participants should be at least 18 years of age.

A total of 133 people accessed the survey, 101 of whom completed the AQ. Of these, 9 did not report their assigned and/or current gender (e.g., responded with “other” or “prefer not to say,”) and 3 reported diagnosed/suspected gender dysphoria/gender identity disorder as well as the same current gender identity as that assigned at birth. These participants were therefore removed from further analysis, leaving a sample of 89. The remaining participants were categorized into one of four groups: (1) assigned female at birth but does not identify as female (transgender AFAB) (*n* = 32), (2) cisgender female (*n* = 21), (3) assigned male at birth but does not identify as male (transgender AMAB) (*n* = 18), or (4) cisgender male (*n* = 18).

### Measures

We used the Autism-Spectrum Quotient (AQ; Baron-Cohen et al., 2001) to quantify autistic traits. The AQ is a 50-item self-report measure of autistic traits that has good test–retest reliability and can differentiate between autistic adults and non-autistic adults (Baron-Cohen et al., 2001, 2014; Woodbury-Smith et al., 2005). The measure includes four response options (“definitely agree,” “slightly agree,” “slightly disagree,” and “definitely disagree”); 26 of the items are reverse scored, and one point is given for each response either slightly or strongly endorsing an autistic trait. Scores range from 0 to 50, with higher scores indicating presence of more traits that are characteristic of autism. Example items include “I prefer to do things the same way over and over again,” and “In a social group, I can easily keep track of several different people’s conversations” (the latter being reverse scored). Internal consistency for the total score in the current study was high (Cronbach’s α = 0.90).

The short forms of the Empathy Quotient (EQ-Short [EQ-S]) and Systemizing Quotient (SQ-Short [SQ-S]) were used to measure empathizing and systemizing, respectively (Wakabayashi et al., 2006). The EQ-S is a 22-item version of the original 60-item EQ (Baron-Cohen & Wheelwright, 2004), and the SQ-S is a 25-item version of the original 60-item SQ (Baron-Cohen et al., 2003). The short forms were used as they are strongly correlated with the full-length scales (Wakabayashi et al., 2006), and because they are quicker to administer. There are four response options for both measures: “strongly agree,” “slightly agree,” “slightly disagree,” and “strongly” disagree.” For the EQ-S, one point is given for a response that slightly endorses an empathizing trait, whereas two points are given for a response that strongly endorses an empathizing trait; for the SQ-S, one point is given for a response that slightly endorses a systemizing trait, and two points are given for a response that strongly endorses a systemizing trait. Example items include “I am good at predicting how someone will feel” (EQ-S), and “I am fascinated by how machines work” (SQ-S). Six items from the EQ-S and 13 items from the SQ-S are reverse-scored. Scores for the EQ-S range from 0 to 44 (higher scores indicating higher empathizing), whereas scores for the SQ-S range from 0 to 50 (higher scores indicating higher systemizing). Internal consistency for the total scores in the current study was high (EQ-S, α = 0.91; SQ-S, α = 0.86). The standardized mean difference (D-score) between empathizing and systemizing was calculated from standardized EQ-S (E) and SQ-S (S) scores as D-score = (S–E)/2.

### Procedure

Participants were informed that the purpose of the study was to assess whether gender identity is related to personality and prenatal testosterone exposure, and that it may help improve our understanding of gender identity as well as individual difference characteristics with which it may be related. We explained that some questions (e.g., gender assigned at birth, current gender identity, ethnicity, sexual orientation, diagnoses of clinical conditions) relate to potentially sensitive topics, and that participants would be free to withdraw from the study at any point without specifying a reason and without penalty. Participants were provided with contact details for the research team, though we additionally stated that we would not be able to inform individuals of their specific questionnaire scores. We also explained that data would be anonymous, that the dataset could be made available online alongside an academic publication, and that participants would be asked to give informed consent if deciding to take part in the study.

After providing informed consent, participants were asked to report their age (18–99 years), gender assigned at birth (“male,” “female,” “other [please specify],”) current gender identity (“male,” “female,” “other [please specify],”) sexual orientation (“heterosexual,” “homosexual,”^1^ “bisexual,” “other [please specify],”) and ethnicity (“Asian/Asian British,” “Black/Black British,” “Black other,” “Chinese,” “Middle/Near Eastern,” “Mixed ethnicity,” “White.”) After this they were administered the AQ, EQ-S, and SQ-S, before reporting whether they had been diagnosed with autism and/or gender dysphoria/gender identity disorder, and whether they suspected they were autistic and/or had gender dysphoria/gender identity disorder. The order of questions was arranged to avoid priming effects, and the information sheet made no explicit reference to autism. Finally, participants were asked to measure the lengths of their second and fourth fingers for a separate study of gender diversity and digit ratio (2D:4D) (see Richards et al., 2020).

The online survey (hosted by Qualtrics) took approximately 20 min to complete. Participants were thanked and debriefed on completion, and signposting information was provided for sources of support for autism and gender identity concerns. Ethical approval was granted by the Faculty of Medical Sciences Research Ethics Committee, Newcastle University (approval number: 1689/12185/2019), and the research was carried out in accordance with the Declaration of Helsinki as revised in 2000.

### Statistical Analysis

The predictor variables were gender assigned at birth (male or female) and current gender (same as assigned at birth or different to assigned at birth), and the outcomes were autistic traits (AQ total score), empathizing (EQ-S total score), systemizing (SQ-S total score), and systemizing-relative-to-empathizing (D-score). AQ, EQ-S, and SQ-S total scores were computed by summing all items from each of the respective scales. We used chi-square tests to ascertain whether autism status (diagnosed/suspected or not diagnosed/suspected) and ethnicity (White or non-White) differed across the four gender groups (transgender AFAB, cisgender female, transgender AMAB, and cisgender male), and whether diagnosed/suspected autism was associated with diagnosed/suspected gender dysphoria/gender identity disorder; the effect size was reported as Cramér’s V and interpreted according to the criteria specified by Cohen (1988) (i.e., 0.10 = small; 0.30 = medium; 0.50 = large). We then used one-way Analysis of Variance (ANOVA) followed by least significant difference tests to determine whether the four gender groups differed significantly in age. We also conducted Pearson’s tests to examine the pattern of intercorrelations between AQ, EQ-S, SQ-S, and D.

As the predictor variables (sex assigned at birth: male or female; current gender: same or different) were categorical and independent, and the outcome variables (AQ, EQ-S, SQ-S, and D) were continuous and approximately normally distributed, we proceeded with a between participants Multivariate Analysis of Covariance (MANCOVA). More specifically, we conducted a MANCOVA followed by individual ANCOVAs for AQ, EQ-S, and SQ-S, but used only ANCOVA when assessing D-scores, as D is derived from two of the other outcome variables (i.e., EQ-S and SQ-S). Age (continuous variable) and autism diagnostic status (diagnosed/suspected autism or not diagnosed/suspected autism) were included as covariates. We included age as a covariate because the age at which a transgender male identity is first reported is typically lower than the age at which a transgender female identity is first reported (Nieder et al., 2011); we included autism status as a covariate so that we could establish whether any observed effects relating to AQ, EQ-S, SQ-S, or D-score were independent of group differences in autism prevalence. Simple effects tests (with Bonferroni adjustment for alpha inflation) were then used to examine for differences between the transgender AFAB group and cisgender female group and between the transgender AMAB group and the cisgender male group. Effect sizes were reported as η_p_^2^ (0.01 = small; 0.06 = medium; 0.14 = large; Cohen, 1988). Data were analyzed using IBM SPSS version 27, and results were considered statistically significant at *p* < 0.05.

## Results

Twenty-two participants in the transgender AFAB group further specified their current gender identity; responses included agender, genderqueer, gender-queer/fluid, non-binary, non-binary or demi-girl, non-binary gender fluid, transmasculine, and transmasculine non-binary. In the transgender AMAB group, three participants further specified their current gender identity; responses included gender fluid and non-binary. Most participants were White, and a wide range of ages and sexual orientations were represented; most gender-diverse participants reported that they found the survey via links from LGBTQ+ support groups, whereas most cisgender participants did not indicate this (see Table 1 for descriptive statistics).

**Table 1:** Demographic information for the whole sample and stratified by gender group

		Transgender AFAB			Cisgender female			Transgender AMAB			Cisgender male		
		*n*	M	SD	*n*	M	SD	*n*	M	SD	*n*	M	SD
Age		32	30.34	11.24	21	33.57	12.91	18	43.50	15.49	18	40.72	17.92

		*n*	%		*n*	%		*n*	%		*n*	%	
Sexual Orientation	Heterosexual	0	0.0%		11	52.4%		4	22.2%		9	50.0%	
	Homosexual	4	12.5%		6	28.6%		4	22.2%		6	33.3%	
	Bisexual	14	43.8%		3	14.3%		4	22.2%		2	11.1%	
	Other/Prefer not to say	14	43.8%		1	4.8%		6	33.3%		1	5.6%	
Ethnicity	White	26	81.3%		16	76.2%		16	88.9%		16	88.9%	
	Not White	6	18.8%		5	23.8%		2	11.1%		2	11.1%	
Autism^1^	Not diagnosed or suspected	18	56.3%		15	71.4%		14	77.8%		17	94.4%	
	Diagnosed	6	18.8%		4	19.0%		1	5.6%		1	5.6%	
	Suspected	9	28.1%		5	23.8%		3	16.7%		1	5.6%	
	Diagnosed and/or suspected	14	43.8%		6	28.6%		4	22.2%		1	5.6%	
Gender Dysphoria^2^	Not diagnosed or suspected	5	15.6%		21	100.0%		2	11.1%		18	100.0%	
	Diagnosed	17	53.1%		0	0.0%		11	61.1%		0	0.0%	
	Suspected	16	50.0%		0	0.0%		9	50.0%		0	0.0%	
	Diagnosed and/or suspected	27	84.4%		0	0.0%		16	88.9%		0	0.0%	
Recruited via LGBTQ+ group?	Yes	17	54.8%		1	5.0%		10	55.6%		6	33.3%	
	No	14	45.2%		19	95.0%		8	44.4%		12	66.7%	

Transgender AFAB = assigned female at birth but does not identify as female; Transgender AMAB = assigned male at birth but does not identify as male

^1^Some participants specified both diagnosed and suspected options for autism; the “diagnosed and/or suspected” category includes all participants who responded in the affirmative to either or both questions

^2^Some participants specified both diagnosed and suspected options for gender dysphoria; the “diagnosed and/or suspected” category includes all participants who responded in the affirmative to either or both questions

### Associations Between Gender Diversity, Autistic Traits, and Empathizing–Systemizing

Means and SD for AQ, EQ-S, SQ-S, and D-scores stratified by gender group are shown in Table 2. A chi-square test revealed the prevalence of diagnosed/suspected autism differed significantly across the four gender groups, χ^2^(3, *N* = 89) = 8.72, *p* = 0.03, V = 0.31. Examination of the observed frequencies compared to those expected by chance alone indicated that there was a relatively high prevalence of autism within the transgender AFAB group and a relatively low prevalence of autism in the cisgender male group. Participants’ ethnicity (White or non-White) did not differ across the four groups, χ^2^(3, *N* = 89) = 1.65, *p* = 0.65, V = 0.14. One-way ANOVA determined that there was a significant difference in participants’ age across the four groups, *F*(3, 85) = 4.32, *p* = 0.01, η_p_^2^ = 0.13. Post hoc least significant difference tests indicated that transgender AFAB participants were younger than transgender AMAB participants (*p* < 0.01) and cisgender males (*p* = 0.01), and that cisgender females were younger than transgender AMAB participants (*p* = 0.03) (all other comparisons were not statistically significant). Interestingly, diagnosed/suspected autism was not significantly associated with diagnosed/suspected gender dysphoria/gender identity disorder, χ^2^(1, *N* = 89) = 1.90, *p* = 0.17, V = 0.15.

**Table 2:** Autistic traits, empathizing, systemizing, and D-scores across gender groups

	AQ			EQ-S			SQ-S			D-score		
	*n*	M	SD	*n*	M	SD	*n*	M	SD	*n*	M	SD
Cisgender female	21	19.43	9.93	19	21.05	10.82	19	13.74	9.68	19	-0.07	0.16
Transgender AFAB	32	25.88	10.25	30	16.87	10.03	29	22.66	9.28	29	0.06	0.16
Cisgender male	18	18.11	7.61	18	20.83	10.00	18	18.94	6.82	18	-0.02	0.15
Transgender AMAB	18	20.17	9.06	18	22.06	9.01	17	21.24	9.54	17	-0.01	0.16

Transgender AFAB = assigned female at birth but does not identify as female; Transgender AMAB = assigned male at birth but does not identify as male; AQ = Autism Spectrum Quotient total score (scores can range from 0 to 50); EQ-S = Empathy Quotient-Short total score (scores can range from = 0 to 44); SQ-S = Systemizing Quotient-Short total score (scores can range from 0 to 50); D-score = difference between standardized EQ-S (E) and standardized SQ-S (S) scores

When examined in the whole sample, the autism-related questionnaire measures showed a pattern of high intercorrelations. AQ was negatively correlated with EQ-S, *r*(83) = − 0.78, *p* < 0.001, and positively correlated with SQ-S, *r*(81) = 0.43, *p* < 0.001, and D-score, *r*(81) = 0.79, *p* < 0.001. EQ-S was weakly negatively correlated with SQ-S, *r*(81) = − 0.22, *p* = 0.04, and D-scores were positively correlated with SQ-S, *r*(81) = 0.73, *p* < 0.001, and negatively correlated with EQ-S, *r*(81) = − 0.83, *p* < 0.001. A similar pattern was observed for each of the four gender identity groups when examined individually (statistical output is not reported here in the interests of brevity).

We used a 2 (gender assigned at birth: male or female) × 2 (current gender: same as assigned or different to assigned) between participants MANCOVA with age and autism status (diagnosed/suspected autism or no diagnosed/suspected autism) as covariates, and AQ, EQ-S, and SQ-S as outcome variables. Box’s *M* test was non-significant, indicating homogeneity of variance–covariance matrices, *M* = 14.15, *p* = 0.79, and Levene’s test was non-significant for each outcome variable (AQ: *F*[3, 79] = 1.44, *p* = 0.24; EQ-S: *F*[3, 79] = 0.23, *p* = 0.88; SQ-S: *F*[3, 79] = 0.79, *p* = 0.50), indicating homogeneity of variances across groups. Although diagnosed/suspected autism was a significant predictor of variance in the outcome variables, Wilks’ λ = 0.58, *F*(3, 75) = 18.19, *p* < 0.001, η_p_^2^ = 0.42, none of the other predictors or covariates were significant: gender assigned at birth, Wilks’ λ = 0.97, *F*(3, 75) = 0.77, *p* = 0.52, η_p_^2^ = 0.03; current gender, Wilks’ λ = 0.92, *F*(3, 75) = 2.06, *p* = 0.11, η_p_^2^ = 0.08; gender assigned at birth × current gender: Wilks’ λ = 0.94, *F*(3, 75) = 1.57, *p* = 0.20, η_p_^2^ = 0.06; age: Wilks’ λ = 0.95, *F*(3, 75) = 1.46, *p* = 0.23, η_p_^2^ = 0.06.

**Fig. 1:**
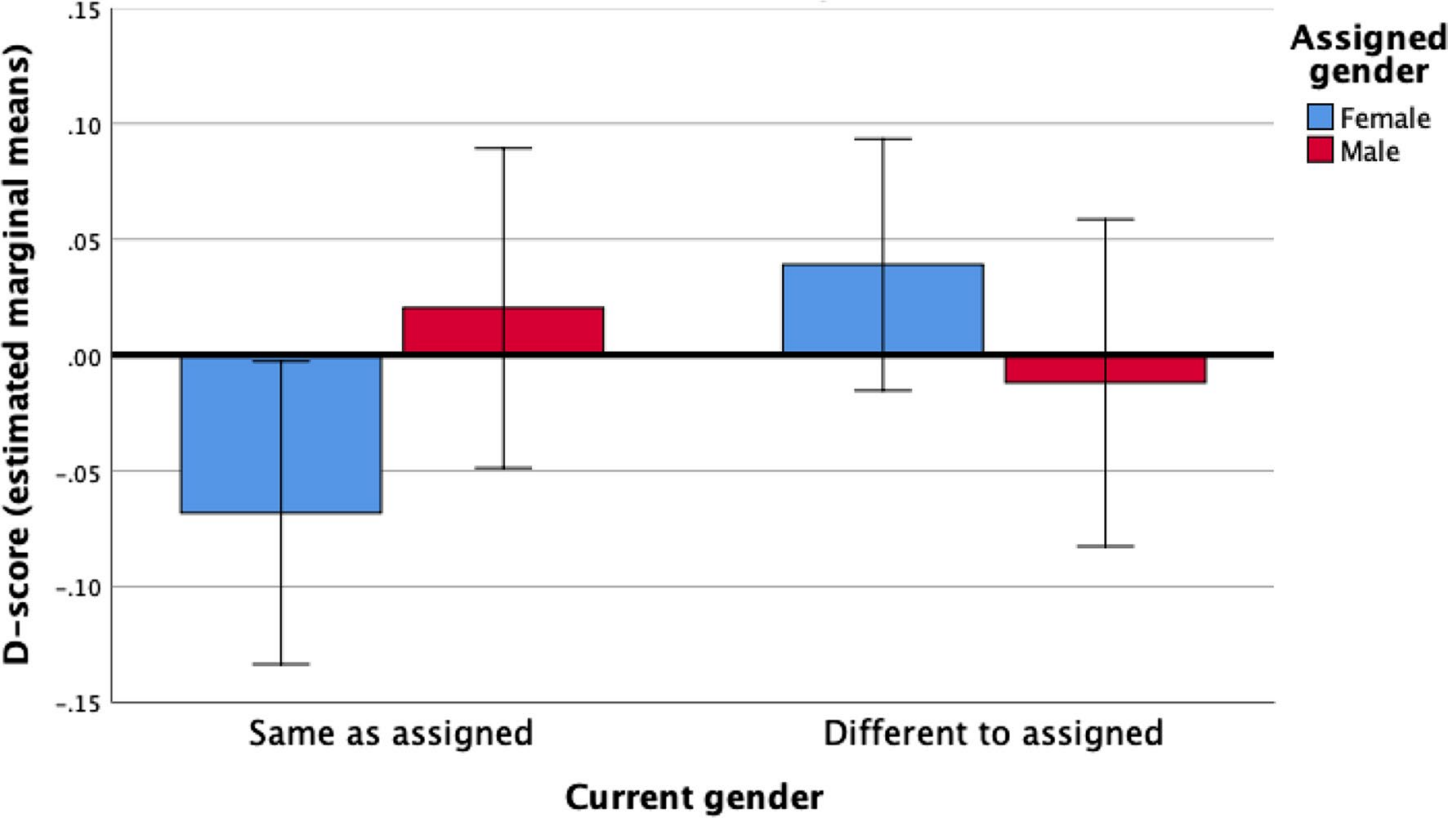
Interaction between gender assigned at birth and current gender on the standardized mean difference between empathizing and systemizing (D-score). Note: High D-scores indicate high systemizing (SQ-S total score) relative to empathizing (EQ-S total score); age and diagnosed/suspected autism were controlled for as covariates; error bars are 95% CI

We elected to follow the MANCOVA with separate ANCOVAs (Table 3). Our reasoning for this was twofold: first, given the relatively small sample, we wanted to avoid excluding participants for whom complete data were not available for all outcome variables, and (2) because we had a priori predictions regarding which groups would differ (and in which direction) for the individual outcome variables. Each ANCOVA model met the assumptions of homogeneity of variance and homogeneity of regression slopes. Diagnosed/suspected autism was a significant predictor of high AQ and low EQ-S scores, whereas having a gender identity different from the sex assigned at birth predicted high SQ-S scores. When we computed a similar ANCOVA model with D-score as the outcome variable, the assigned gender × current gender interaction term was significant (see Fig. 1).

**Table 3:** Outcome of ANCOVA models with assigned gender, current gender, and the assigned × current gender interaction term as predictors, autism status and age as covariates, and AQ, EQ-S, SQ-S, and D-score as outcomes

	AQ			EQ-S			SQ-S			D-score		
	F	*p*	η_p_^2^	F	*p*	η_p_^2^	F	*p*	η_p_^2^	F	*p*	η_p_^2^
Assigned gender	0.65	0.42	0.01	< 0.01	0.98	< 0.001	0.83	0.37	0.01	0.30	0.59	< 0.01
Current gender	1.57	0.21	0.02	0.06	0.81	< 0.01	5.56	0.02	0.07	1.32	0.26	0.02
Assigned × current gender	2.28	0.14	0.03	2.23	0.14	0.03	3.05	0.09	0.04	4.78	0.03	0.06
Age	2.07	0.15	0.02	0.43	0.51	0.01	1.38	0.24	0.02	1.70	0.20	0.02
Autism status	43.38	< 0.001	0.34	27.20	< 0.001	0.26	2.64	0.11	0.03	22.51	< 0.001	0.23

Degrees of freedom for each ANCOVA model are as follow: AQ (1, 83); EQ-S (1, 79); SQ-S (1, 77); D-score (1, 77)

We used simple effects tests with Bonferroni correction (required α: *p* < 0.025) to determine whether the adjusted means for each outcome variable differed between the transgender AFAB group and the cisgender female group, and whether they differed between the transgender AMAB group and the cisgender male group. Although the transgender AFAB and cisgender female groups did not differ for EQ-S (*F*[1, 79] = 0.88, *p* = 0.35, η_p_^2^ = 0.01), higher scores were observed in the transgender AFAB group for AQ (*F*[1, 83] = 4.61, *p* = 0.04, η_p_^2^ = 0.05), SQ-S (*F*[1, 77] = 9.84, *p* < 0.01, η_p_^2^ = 0.11), and D (*F*[1, 77] = 6.42, *p* = 0.01, η_p_^2^ = 0.08) (though note that the effect for AQ did not survive Bonferroni correction). There were no significant differences between the transgender AMAB group and the cisgender male group for any of the outcomes examined: AQ, *F*(1, 83) = 0.03, *p* = 0.87, η_p_^2^ < 0.001; EQ-S, *F*(1, 79) = 1.32, *p* = 0.26, η_p_^2^ = 0.02; SQ-S, *F*(1, 77) = 0.19, *p* = 0.67, η_p_^2^ < 0.01; D, *F*(1, 77) = 0.44, *p* = 0.51, η_p_^2^ = 0.01.

## Discussion

The current study examined autistic traits, empathizing, and systemizing in an adult sample representing a broad range of gender identities and sexual orientations. In line with our predictions, there were no significant differences between the transgender AMAB and cisgender male groups but AQ, SQ-S, and D-scores were higher, and EQ-S scores were lower, in transgender AFAB participants than cisgender female participants. Although the effect for EQ-S was not significant, and that relating to AQ did not survive Bonferroni correction, the overall pattern is consistent with predictions derived from the EMB theory (see Jones et al., 2012). It should be noted, however, that the transgender AFAB group was the only one for which the mean AQ score (*M* = 25.88, *SD* = 10.25) approached the suggested cut-off points of 26 (Woodbury-Smith et al., 2005) or 32 (Baron-Cohen et al., 2001) that may indicate clinical concern, and that this group also included the highest prevalence of diagnosed and/or suspected autism (43.8%). However, as less than half (6 of 14) of those who indicated diagnosed and/or suspected autism reported that they had received a diagnosis, this pattern of results remains consistent with the notion that there is an elevated prevalence of undiagnosed autism associated with transgender AFAB status.

Although the current findings align with those of research linking gender diversity with autism/autistic traits, potential explanations for this co-occurrence remain speculative (for an overview, see van der Miesen et al., 2016). The EMB theory, which suggests that autism represents a hyper-masculinized cognitive profile (i.e., systemizing >  > empathizing) (Baron-Cohen, 2002, 2003; Baron-Cohen et al., 2005; Greenberg et al., 2018), implies that gender diversity in autistic birth-assigned females could be an expression of an “extreme male” characteristic. Consistent with this theory is the idea that autism and gender diversity in birth-assigned females share a biological underpinning in terms of atypical fetal sex hormone exposure. Evidence for this comes from studies showing that second trimester amniotic testosterone levels correlate positively with autistic traits (Auyeung et al., 2009, 2010, 2012; though see also Kung et al., 2016, who did not replicate this effect) and systemizing (Auyeung et al., 2006) and negatively with empathizing (Chapman et al., 2006), and that elevated levels of androgens (Baron-Cohen et al., 2015) and estrogens (Baron-Cohen et al., 2020) are present in the amniotic fluid of males who develop autism.

The finding of the current study that elevated autistic traits, systemizing, and systemizing-relative-to-empathizing were present in the transgender AFAB group but not in the transgender AMAB group is consistent with predictions of the EMB theory, as are findings of studies that report a stronger association between autism and gender diversity in birth-assigned females than birth-assigned males (Cooper et al., 2018; Dewinter et al., 2017; Jones et al., 2012; Kung, 2020; van der Miesen et al., 2018 [adolescent sample]; see also Nobili et al., 2018; Vermaat et al., 2018). However, other studies have reported no significant sex difference (e.g., Hisle-Gorman et al., 2019; Pasterski et al., 2014; Skagerberg et al., 2015; Strang et al., 2014; van der Miesen et al., 2018 [adult sample]) or even a higher prevalence of autism in birth-assigned males presenting at gender clinics (de Vries et al., 2010; Heylens et al., 2018). Considering the equivocal pattern of results in this area, future studies utilizing large sample sizes should examine differences in prevalence of autism diagnosis, autistic traits, and empathizing and systemizing across transgender AFAB, transgender AMAB, cisgender female, and cisgender male groups. One such possibility would be to examine data from the BBC Internet Study (see Reimers, 2007), as participants completed self-report measures of empathizing and systemizing (Manning et al., 2010a, 2010b) and also reported their birth-assigned sex as well as gender identity (Manning et al., 2020). Additionally, it could be useful to examine the question meta-analytically (Warrier et al., 2020).

It should be noted that the EMB theory does not explain why autistic males report higher rates of gender dysphoria relative to typically developing males (van der Miesen et al., 2016), and a recent trend for birth-assigned females to be more likely than birth-assigned males to present at gender clinics (Aitken et al., 2015) may suggest a broader societal phenomenon rather than one that can be explained in terms of differences in prenatal testosterone levels (van der Miesen et al., 2018). Furthermore, there may be reasons other than those biological for explaining sex differences in autism diagnosis: for instance, it has been noted that diagnostic algorithms were developed based on predominantly male samples, and so may not be sensitive to detecting autism in females and gender-diverse individuals (Ratto, 2021), which can lead to a diagnostic bias in favor of males (Loomes et al., 2017). It is also acknowledged that some autistic adults with transgender or gender-diverse identities report finding terminology related to the EMB theory unhelpful, and suggest that the notion of being categorized as having an extreme male brain could increase their sense of dysphoria (Coleman-Smith et al., 2020). Future work should investigate this consideration and aim to determine improved ways of supporting this population.

Although biological factors are clearly important in the development of one’s gender identity (Hines, 2004; Meyer-Bahlburg, 2005), different expectations of males and females as regards adherence to socially derived gender roles should not be overlooked (George & Stokes, 2018; Kanfiszer et al., 2017). Notably, autistic females report higher masculinity and lower femininity than non-autistic females (Cooper et al., 2018), often prefer socializing with males than females (Bargiela et al., 2016), report elevated rates of tomboyism in childhood and adolescence (Bejerot & Eriksson, 2014; Ingudomnukul et al., 2007), and may not automatically identify with the construct of femininity as strongly as non-autistic females do (Kanfiszer et al., 2017). However, it remains unclear whether such factors are causes and/or consequences (or merely correlates) of gender diversity, and indeed autistic features (e.g., intense/obsessional interests; Landén & Rasmussen, 1997; Zucker et al., 2017) could be mistaken for gender dysphoria symptoms and vice versa (Heylens et al., 2018). Additionally, other social processes (e.g., social and communication difficulties associated with autism; Landén & Rasmussen [1997]) may play important roles in explaining the link between autism and gender dysphoria. This suggestion is supported by George and Stokes' (2018) finding that gender dysphoria traits were most strongly associated with the Social Skill (*r* = 0.35) and Communication (*r* = 0.36) subscales of the AQ (although a comparably sized correlation was also observed for cognitive inflexibility [*r* = 0.34]). However, it should be noted that self-report measures of autistic traits have not been validated in gender-diverse populations, that they may lack specificity, and that elevated scores on such measures could reflect social difficulties (e.g., challenges in relating to peers) associated with gender dysphoria rather than autism (Heylens et al., 2018; Skagerberg et al., 2015).

A strength of the current research is that we sampled a wide range of gender identities and sexual orientations (Ratto, 2021), although it should be acknowledged that individuals who are members of support groups are unlikely to be truly representative of autistic and/or transgender and gender-diverse people. This consideration might be reflected in the observation that not only were the rates of diagnosed/suspected autism higher than the general adult population average (approximately 1–2%; Brugha et al., 2016) in the transgender AFAB (43.8%) and transgender AMAB (22.2%) groups, but they were also considerably higher than might be expected in the cisgender female (28.6%) and cisgender male (5.6%) groups. It is unclear why this is the case, as we deliberately did not approach groups that related specifically to autism and avoided referring to autism when advertising the study on social media. However, as a considerable proportion of our participants found the study via links from LGBTQ+ support groups, it is possible that this higher prevalence of autism within our sample relies, at least in part, on the observation that there is greater variability in the sexual orientation of autistic adults compared to non-autistic adults (Bejerot & Eriksson, 2014; Cooper et al., 2018; Dewinter et al., 2017). Unfortunately, the terminology we used regarding sexual orientation (heterosexual, homosexual, etc.) can create confusion in the current context, particularly as we did not specify whether this should relate to one’s sex assigned at birth or one’s current gender identity; it would therefore have been more informative to use less ambiguous terms such as “gynophilic” and “androphilic.” Furthermore, as no participants in the transgender AFAB group in our study reported that they were heterosexual, it was not possible to reliably determine whether the effects observed here relate to gender identity, sexual orientation, or both. This is an important limitation considering that Vermaat et al. (2018) found that AFAB participants with non-heterosexual orientations in their sample reported the highest levels of autistic traits, and also because George and Stokes (2018) observed that gender dysphoria traits were a significant mediator of the association between AQ score and sexual orientation. Furthermore, it is currently unknown whether autistic traits are qualitatively different in gender-diverse individuals compared to cisgender individuals, and this needs to be investigated. This is particularly important considering that those with social anxiety disorder, on average, obtain higher AQ scores than population controls (Hoekstra et al., 2008), and anxiety disorders are frequently present in both autistic (Hollocks et al., 2019) and gender-diverse people (Millet et al., 2017).

A further limitation of the current study is that the relatively small sample size necessitated the use of a binary (cisgender/non-cisgender) approach to data analysis that does not consider the extensive variation that is present. Unfortunately, our method for recruiting participants made it unfeasible to obtain a larger sample. Whereas a similar online study (Kristensen & Broome, 2015) recruited a larger number of participants (included in main analysis: *n* = 446) through snowball sampling, we contacted every LGBTQ+ support group in the UK of which we were aware, and so could not add more participants without contacting organizations from elsewhere (which could increase the heterogeneity of our sample in unpredictable ways). Although this is a limitation, the fact that we still observed statistically significant differences consistent with those already reported in the literature provides evidence that these effects are robust. It should, however, also be acknowledged that we did not assess participants’ hormonal treatment status. This may be a particularly important oversight because, although Nobili et al. (2020) reported that autistic traits remain stable following cross-sex hormone treatment, they also found that transgender AFAB adults were more likely than transgender AMAB adults to report clinically significant levels of autistic traits post-treatment even though there was no such difference at baseline.

### Implications

Research into this area is important because a greater understanding of the association between autism and transgender and gender-diverse identities could improve processes relating to detection, diagnosis, and support. Furthermore, there is much that we do not know about how autistic traits impact the support needs of gender-diverse individuals. Lack of understanding of one’s own and others’ emotions, problems interpreting social cues and inability to effectively communicate one’s thoughts are just some of many obstacles that may adversely interfere with therapeutic gender treatments in those with autistic features (Glidden et al., 2016; Jacobs et al., 2014). As has been suggested by others (e.g., George & Stokes, 2018), it is therefore vital to establish an improved understanding of core autistic traits present in those who are both autistic and gender diverse, as this could help provide each person with the most effective support for their specific needs.

Taken together, the findings of the current study are broadly in line with predictions derived from the EMB theory of autism. However, given the unique challenges faced by those who are both autistic and gender diverse (Strang, Powers, et al., 2018) further research in this area is needed regardless of what the biological, cultural, and/or societal antecedents might be.

## Data Availability

The dataset and SPSS syntax file supporting this article are available on the Open Science Framework: https://osf.io/jzc6w/.

## Footnotes

1 We acknowledge that the American Psychological Association (2020, p. 147) advises against using the term "homosexual," though do so here for accuracy, as this is the terminology that was used in the online survey administered to the participants of the current study.
